# Late-Onset *N*-Acetylglutamate Synthase Deficiency: Report of a Paradigmatic Adult Case Presenting with Headaches and Review of the Literature

**DOI:** 10.3390/ijms19020345

**Published:** 2018-01-24

**Authors:** Catia Cavicchi, Chiara Chilleri, Antonella Fioravanti, Lorenzo Ferri, Francesco Ripandelli, Cinzia Costa, Paolo Calabresi, Paolo Prontera, Francesca Pochiero, Elisabetta Pasquini, Silvia Funghini, Giancarlo la Marca, Maria Alice Donati, Amelia Morrone

**Affiliations:** 1 Molecular and Cell Biology Laboratory of Neurometabolic Diseases, Neuroscience Department, Meyer Children’s Hospital, 50139 Florence, Italy; catia.cavicchi@meyer.it (C.C.); chiara.chilleri@meyer.it (C.C.); lorenzo.ferri@meyer.it (L.F.); 2 Structural Biology Researcher Center, VIB, Vrije Universiteit Brussel, 1050 Brussels, Belgium; Antonella.Fioravanti@vub.be; 3 Neurology Unit, Santa Maria della Misericordia Hospital, 06123 Perugia, Italy; fra.ripandelli@gmail.com (F.R.); cinzia.costa@unipg.it (C.C.); paolo.calabresi@unipg.it (P.C.); 4 Medical Genetics Unit, Santa Maria della Misericordia Hospital, 06123 Perugia, Italy; pprontera@hotmail.com; 5 Metabolic and Muscular Unit, Neuroscience Department, Meyer Children’s Hospital, 50139 Florence, Italy; francesca.pochiero@meyer.it (F.P.); elisabetta.pasquini@meyer.it (E.P.); m.donati@meyer.it (M.A.D.); 6 Newborn Screening, Biochemistry and Pharmacology Laboratory, Neuroscience Department, Meyer Children’s Hospital, 50139 Florence, Italy; silvia.funghini@meyer.it (S.F.); g.lamarca@meyer.it (G.l.M.); 7 Department of Experimental and Clinical Biomedical Sciences, University of Florence, 50139 Florence, Italy; 8 Department of Neurosciences, Psychology, Drug Research and Child Health (NEUROFARBA), University of Florence, 50139 Florence, Italy

**Keywords:** *N*-acetylglutamate synthase deficiency (NAGSD), *NAGS* gene mutations, *N*-carbamylglutamate (NCG), urea cycle disorders (UCDs), late-onset UCDs, hyperammonemic encephalopathy, headaches

## Abstract

*N*-acetylglutamate synthase deficiency (NAGSD) is an extremely rare urea cycle disorder (UCD) with few adult cases so far described. Diagnosis of late-onset presentations is difficult and delayed treatment may increase the risk of severe hyperammonemia. We describe a 52-year-old woman with recurrent headaches who experienced an acute onset of NAGSD. As very few papers focus on headaches in UCDs, we also report a literature review of types and pathophysiologic mechanisms of UCD-related headaches. In our case, headaches had been present since puberty (3–4 days a week) and were often accompanied by nausea, vomiting, or behavioural changes. Despite three previous episodes of altered consciousness, ammonia was measured for the first time at 52 years and levels were increased. Identification of the new homozygous c.344C>T (p.Ala115Val) *NAGS* variant allowed the definite diagnosis of NAGSD. Bioinformatic analysis suggested that an order/disorder alteration of the mutated form could affect the arginine-binding site, resulting in poor enzyme activation and late-onset presentation. After optimized treatment for NAGSD, ammonia and amino acid levels were constantly normal and prevented other headache bouts. The manuscript underlies that headache may be the presenting symptom of UCDs and provides clues for the rapid diagnosis and treatment of late-onset NAGSD.

## 1. Introduction

Inherited *N*-acetylglutamate synthase deficiency (NAGSD, #237310) is an extremely rare urea cycle disorder (UCD) (estimated incidence: less than 1:2,000,000) caused by recessive mutations in the *NAGS* gene [1]. To date, 45 *NAGS* mutations have been reported, of which 60% are missense mutations [2,3]. Mitochondrial *N*-acetylglutamate synthase (NAGS; EC 2.3.1.1) synthesizes the allosteric activator of the first enzyme of the urea cycle, carbamoylphosphate synthetase 1 (CPS1; EC 6.3.4.16) [4]. The human NAGS (hNAGS) enzyme is organized into an *N*-terminal amino acid kinase (AAK) domain (residues 95–372) containing the l-arginine binding site and a C-terminal *N*-acetyltransferase (NAT) domain [5]. Clinically and biochemically, NAGSD is indistinguishable from CPS1 deficiency (CPS1D), and common biochemical features include increased amounts of plasma ammonia and glutamine, reduced plasma citrulline, and normal or low levels of urinary orotic acid [6]. NAGS enzyme assays are problematic as they necessitate a liver specimen and so molecular analysis is the gold standard test for confirming NAGSD.

Adult patients are very rare and often unrecognized. They may present with a self-selected low protein diet, headaches, vomiting, lethargy, behavioural changes, confusion, episodes of altered consciousness, and hyperammonemic encephalopathy [7,8]. Differential diagnosis between NAGSD and other UCDs is important, as a specific and effective treatment for NAGSD using *N*-carbamylglutamate, a structural analogue of the natural CPS1 activator (NCG; carglumic acid; Carbaglu, Orphan Europe, Paris France), is available [9].

Here we report a 52-year-old woman with a history of recurrent headaches who developed an acute onset of NAGSD. As headaches may be an early and unspecified symptom of UCDs, we also performed a literature review of UCD-related headaches focusing on types and pathophysiologic mechanisms, contextualizing the latter within the International Classification of Headache Disorders 3rd edition, beta version (ICHD-3 beta) [10].

## 2. Results

### 2.1. Case Report

The patient was a 52-year-old Italian woman with consanguineous healthy parents. Her medical history revealed recurrent headaches since puberty (Table S1) and three previous acute episodes of altered consciousness, which resolved spontaneously. Two acute crises occurred at 20 and 24 years of age, both in postpartum periods on the fourth day after delivery, when the patient manifested psychomotor agitation, anxiety, and confusion. During the first episode, she also experienced severe visual impairment, accompanied by confusion and anxiety in the few hours after she was discharged from the Maternity Unit. For 10 days, she showed behaviour and mood changes and required sedation. After a 20-day hospitalization, a diagnosis of postpartum psychosis was made. During the second postpartum crisis, symptoms included disorientation, confusion, and mydriasis, causing a new 10-day hospital admission. 

The third crisis occurred at 51 years of age when the patient suddenly became confused. She was admitted to the Emergency Department because of marked coordination difficulties, disorientation, and anxiety. Within a few hours, she also experienced severe headache, nausea, and vomiting. Video electroencephalogram (EEG) monitoring showed bilateral frontal epileptiform and slow activity. Brain MRI showed hyperintensities in the cortical frontal regions. A CADASIL (Cerebral autosomal dominant arteriopathy with subcortical infarcts and leukoencephalopathy; OMIM #125310) was suspected but *NOTCH3* gene testing was negative. Neurological and behavioural abnormalities spontaneously disappeared after a few days.

At 52 years of age she was admitted to the Neurology Unit with clinical features similar to the last episode. Her husband reported that she performed repetitive and senseless actions, such as undressing and dressing again or opening and closing the dishwasher. This crisis was preceded by a period of insomnia, severe headaches, low caloric intake, and dehydration. She reported she had never enjoyed meat and self-selected a low protein diet. Ammonia level on admission was 45 µmol/L (normal values:(n.v.) 11–32), but rapidly increased to a maximum of 330 µmol/L. EEG showed abnormalities which were consistent with non-convulsive status epilepticus. During the hospitalization, hyperammonemia associated with confusion, psychomotor agitation (i.e., echolalia and motor stereotypies), nausea, and vomiting was detected several times, mainly in the evening. Suspecting a UCD, protein intake was stopped and glucose intravenous infusion and oral supplementation with l-arginine (195 mg/kg/day) were started. Subsequently a low protein diet was introduced. Unfortunately, metabolic investigations were performed on samples collected after the initiation of arginine therapy and were inconclusive (Table S2). The patient’s clinical status gradually improved with normalization of plasma ammonia levels. However, ammonia concentrations fluctuated considerably over the follow-up period and sodium benzoate 160 mg/kg/day in 4 divided doses was introduced. After molecular confirmation of NAGSD, NCG treatment 16 mg/kg/day in 4 doses was started. Sodium benzoate was gradually reduced and then discontinued, as was the l-arginine. She did not experience further episodes of altered consciousness and headaches. She is currently treated with NCG 24 mg/kg/day. After 6 months of NCG treatment, plasma ammonia and amino acid levels are constantly normal. 

### 2.2. Molecular Investigation

Molecular testing of the *OTC* and *CPS1* genes came back negative. Sequence analysis of the *NAGS* gene identified, in the final part of exon 1, the homozygous c.344C>T variant which replaces alanine 115 of the NAGS protein with a valine (p.Ala115Val) (reference sequences: NM_153006.2 and NP_694551). Such a variant was not reported in the Human Gene Mutation Database (HGMD) professional (http://www.biobase-international.com/product/hgmd, accessed on 20 September 2017) and in the Genome Aggregation Database (gnomAD) (http://gnomad.broadinstitute.org, accessed on 20 September 2017), and was detected in a heterozygous state in both unaffected parents of the patient.

Alamut Visual software (http://www.interactive-biosoftware.com/alamut-visual/, accessed on 20 September 2017) suggested two possible pathogenic mechanisms for the c.344C>T (p.Ala115Val) variant: damage to NAGS protein function due to the missense change and a splicing defect due to the creation of a new donor splice site. PolyPhen-2 (http://genetics.bwh.harvard.edu/pph2/, accessed on 20 September 2017) and MutationTaster (http://www.mutationtaster.org/, accessed on 20 September 2017) tools classified the p.Ala115Val amino acid substitution as pathogenic.

**Figure 1:**
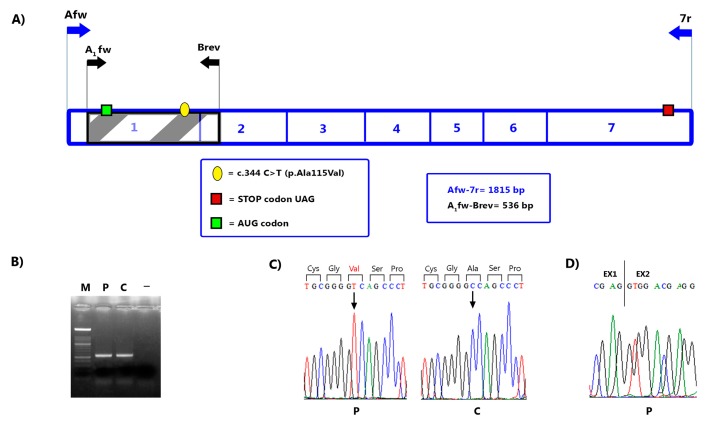
Analysis of *NAGS* RNA. (**A**) Schematic representation of *NAGS* cDNA. Numbers in the cDNA diagram show the *NAGS* exons (1–7). Primers used for RT-PCR analysis are indicated by arrows: the blue primers (Afw and 7r) were used to amplified the entire coding region of the *NAGS* gene and the black primers (A_1_fw and Brev) the fragment containing the c.344C>T (p.Ala115Val) variant. The hatched bar highlights the region of interest encompassing exons 1 and 2 and the new variant c.344C>T (p.Ala115Val). (**B**) RT-PCR products of the fragment A_1_fw-Brev including part of the exons 1 and 2. Abbreviations: M, molecular marker; P, patient; C, normal control; -, no template cDNA. (**C**) Partial nucleotide sequence of the *NAGS* cDNA region including the variant c.344C>T (p.Ala115Val). Vertical arrows indicate the position of the mutated nucleotide in the patient and the corresponding one in the normal control. The amino acid sequence is indicated in the upper line and the mutated residue in the patient is coloured in red. (**D**) Partial nucleotide sequence of the junction between exons 1 and 2 in the patient cDNA. The electropherogram shows conservation of the physiological exon junction.

*NAGS* RNA analysis confirmed the presence of the c.344C>T substitution at a homozygous level but it also demonstrated conservation of the physiological junction between exons 1 and 2, thus excluding a splicing defect (Figure 1).

**Figure 2:**
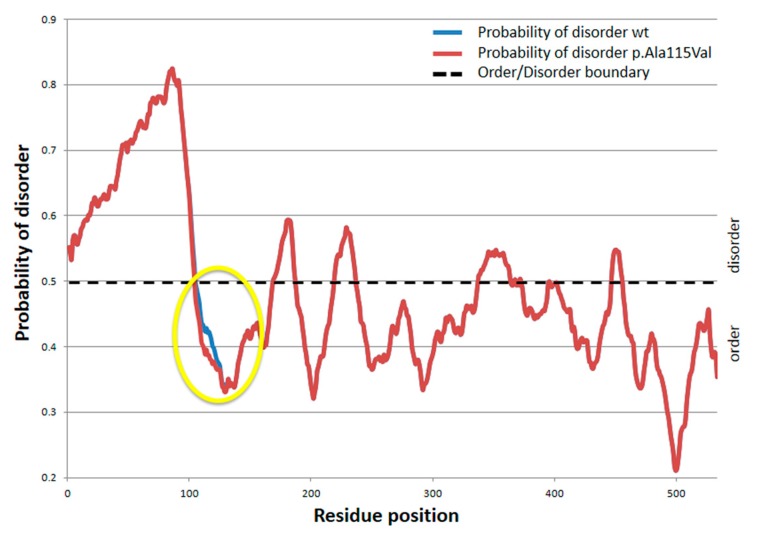
Per-residue disordered prediction of wild-type NAGS protein versus the p.Ala115Val mutated form. The wild type (in blue) and p.Ala115Val variant (in red) amino acid NAGS sequences were analysed by RONN software. For each amino acid, the probability of disorder score has been assigned. The black dotted line indicates the order/disorder boundary. The p.Ala115Val substitution occurs in a region of the protein that is estimated by RONN analysis to be below the order/disorder boundary, in the ordered predicted area. The single amino acid substitution p.Ala115Val results in a decrease in the probability of disorder that affects the mutated amino acid and its neighbouring region (between residues 93 and 133, highlighted in the figure in the yellow circle).

The p.Ala115Val mutation affects a residue located in the AAK domain. Since the crystal structure of the domain is not yet available, we cannot map the variant in the structural model; however, we used Regional Order Neural Network (RONN) software to predict the potential effect of p.Ala115Val on the protein secondary structure. Comparison of structural disorder between wild-type and p.Ala115Val mutant proteins showed that p.Ala115Val increases the probability that such a protein region becomes more ordered (Figure 2). 

This hypothesis is supported by results obtained from several secondary structure prediction tools [3], which localized the p.Ala115Val variant in a short coil/turn region that connects secondary structure elements of the protein. According to such predictions, the *NAGS* variant is shortening the coil/turn region while extending the neighboring secondary structure elements. This change could induce stretching of the surrounding secondary structure elements, causing a gain in rigidity in the 3D AKK domain structure.

## 3. Discussion 

Although UCD onset traditionally occurs in paediatric-age patients, there is considerable evidence that cases recognized in adulthood are increasing [11,12]. For NAGSD, to the best of our knowledge, only six cases have been diagnosed over 30 years of age: a 33-year-old female with altered mental status and coma after caesarean section [13]; a 33-year-old female with seizures and coma during pregnancy [14,15]; a 33-year-old male with post-operative combativeness, confusion, and seizures [13]; a 38-year-old male with confusion, nausea, and vomiting [7]; a 57-year-old female with migraine headaches, staring spells, and coma [16]; and finally a 59-year-old female with hyperammonemic coma after pelvic fracture [17]. In all these reported adult cases, plasma ammonia levels were found to be markedly elevated.

The clinical signs and symptoms of late-onset UCDs may be subtle, unspecific, and episodic, and clinical status may sometimes normalise, especially if the patient self-selects a low-protein diet [8]. For these reasons, diagnosis may be difficult and delayed. Our case endured a lifelong “diagnostic odyssey” and her misdiagnoses included primary headache, postpartum psychosis, and CADASIL. During the acute episodes, the clinical features of NAGSD in our patient included: gastrointestinal symptoms (nausea and vomiting), psychiatric symptoms (psychomotor agitation, anxiety, behaviour and mood changes), and neurological symptoms (unexplained episodes of altered consciousness, coordination difficulties, complete visual loss, mydriasis, and headache).

Our patient had suffered from recurrent headaches since puberty. Headaches were often accompanied by nausea, vomiting, or behavioural changes, but they were not precisely classified and associated with a UCD. So far, three types of headache have been reported in UCDs: (a) migraine-associated cyclic vomiting syndrome [18], (b) sporadic hemiplegic migraine in OTC deficiency (OTCD) [19], and (c) tension-type headache in citrullinemia type II [20]. Headache pathophysiology in UCDs has not yet been entirely elucidated, however headaches in hyperammonemic patients may be correlated with brain ammonia toxicity, resulting in osmotic cell swelling and increased intracranial pressure from rapid intracellular accumulation of glutamine [21]. According to the ICHD-3 beta, such headaches belong to the category of “headaches attributed to intracranial hypertension” (i.e., “non-vascular intracranial disorder”). In non-hyperammonemic patients affected by proximal UCDs, nitric oxid (NO) may have a role in headache pathophysiology. It has been reported that in some OTCD carriers manifesting headaches, oral l-arginine administration reduces the severity and occurrence of headaches by restoring blood NO levels [22]. Since NO acts as a vasodilator, low NO synthesis due to low arginine levels causes vasospasm that might precipitate headaches. Hence, headaches in non-hyperammonemic UCD patients belong to the category “headaches attributed to cranial or cervical vascular disorder”. Since in our patient headaches were present throughout her lifetime and some severe bouts coincided with altered consciousness, it is likely that both NO abnormalities and hyperammonemia contributed. The patient did not experience any headaches after NAGSD diagnosis and specific therapy, demonstrating that normal arginine and ammonia levels prevented the manifestation of headaches. Since puberty, headaches in our patient were partially controlled using non-steroidal anti-inflammatory drugs (NSAIDs) (acetylsalicylic acid and ibuprofen), which gave transient pain relief via inhibition of the cyclooxygenase enzymes. Obviously, these drugs were not able to resolve the major symptoms and metabolic abnormalities of NAGSD, and headache bouts recurred after the NSAIDs were suspended. Moreover, these drugs can cause adverse reactions in the gastrointestinal system [23]. In comparison, treatment with NCG is specific for NAGSD and in our patient had long-term effectiveness in treating headache pain, without significant undesirable effects.

UCD patients have a high risk of developing hyperammonemia, especially after exposure to precipitating environmental or stress factors [6,12]. In our patient, ammonia measurement was only performed at 52 years of age during the last crisis after a period of low caloric intake and dehydration. Peak levels of hyperammonemia were detected especially after meal consumption. Protein load and catabolic events are known to be important precipitants for UCDs [8,12]. In two previous crises in our patient the precipitant was the puerperium, which is characterized by involution of the uterus and increased catabolism [8]. It is remarkable that during the postpartum crises she experienced complete visual loss and mydriasis. Although ammonia was not measured at that time, these ocular symptoms are likely to be correlated with hyperammonemia. In our experience, visual impairment is also present during acute severe hyperammonemia of late-onset CPS1D [24] and OCTD. Although acute symptomatology in our patient mainly occurred due to diet and catabolic stress, we cannot exclude a contribution of the gut microbiota in the progression and severity of the disease. It is known that in gut bacteria, urease converts urea produced by the liver as a waste product to ammonia and carbon dioxide, contributing to hyperammonemia-associated neurotoxicity [25]. Thus, reduction of gut urease activity could be an adjuvant therapeutic target in late-onset UCD cases when current treatment for hyperammonemia is suboptimal. A clinical trial using the urease inhibitor acetohydroxamic acid is currently underway in adults with UCD at the Children’s National Medical Center, Washington D.C., District of Columbia, United States (ClinicalTrials.gov Identifier: NCT03181828).

Diagnosis of UCD in our patient was difficult, because amino acid and orotic acid assays were only performed after the initiation of arginine therapy. At first, we performed genetic testing for X-linked OTCD because parental consanguinity was not reported at that time and because this defect is the most frequent UCD and females may be manifesting carriers. Then we analyzed the *CPS1* and *NAGS* genes, both responsible for autosomal recessive UCDs. Molecular analysis of the *NAGS* gene identified the new homozygous c.344C>T (p.Ala115Val) variant both on genomic DNA and cDNA and excluded a splicing defect due to such a variant. 

RONN software predicted that the p.Ala115Val variant causes a localized order alteration which could preserve the protein folding but influence the interactions between protein domains. The p.Ala115 residue is located close to the inferred-arginine binding residue [3]. The binding of arginine to the AAK domain normally induces conformational changes that increase the affinity of NAGS to bind its substrate [26]. Hence, the p.Ala115Val variant could compromise the correct binding of arginine, limiting but not preventing the interaction between AAK and NAT domains, and may finally result in poor enzyme activation. This pathophysiologic mechanism leads to a partial enzymatic deficiency and hence correlates with a late-onset NAGSD. Correlation between the p.Ala115Val variant and late-onset NAGSD is corroborated by the fact that missense mutations in the AAK domain are usually responsible for milder phenotypes than mutations in the NAT domain [15]. A recent mutational study of the *NAGS* gene reported a null frequency of missense mutations for exon 1, which encodes the mitochondrial signal peptide, the variable domain, and a slight part of the AAK domain [3]. Hence, p.Ala115Val is the first missense variant with clinical consequences mapping in exon 1 of the *NAGS* gene. Our patient is homozygous for the c.344C>T (p.Ala115Val) variant. This finding is in line with recent literature, since about 75% of NAGSD patients are homozygous [2,3]. The high homozygosity rate depends on founder effects due to endogamy and inbreeding, which increase the genetic risk for rare recessive disorders, such as NAGSD.

## 4. Materials and Methods

The patient and her parents gave their written informed consent for inclusion in the study. The study was conducted in accordance with the Declaration of Helsinki, and the protocol was approved by the Ethics Committee of the Tuscany Region (Project identification code: CEP012018, Date: 16/1/2018).

Genomic DNA was isolated from peripheral blood using a QIAsymphony instrument (Qiagen, Hilden, Germany). Molecular analysis, including the *OTC*, *CPS1,* and *NAGS* genes, was performed by Sanger sequencing of the coding region and intron–exon boundaries using an ABI PRISM 3130 Genetic Analyser (Applied Biosystems, Foster City, CA, USA). *OTC* and *NAGS* genes were also screened for the previously reported deep intronic and regulatory mutations [27,28,29]. Large deletion/duplication of the *OTC* gene was investigated by multiplex ligation-dependent probe amplification (MLPA) using the SALSA MLPA probemix P079-A3 *OTC* kit (MRC-Holland, Amsterdam, The Netherlands). 

The novelty of the *NAGS* c.344C>T (p.Ala115Val) variant was examined using the HGMD professional and the gnomAD databases. Its pathogenicity was predicted with Alamut Visual software and the effect of the p.Ala115Val substitution on the native structural order/disorder of the NAGS protein was evaluated by RONN software [30]. 

To investigate a possible splicing defect caused by the new *NAGS* variant, we performed reverse transcription polymerase chain reaction (RT-PCR) assays on the patient’s RNA, extracted from cultured lymphocytes. Total RNA was obtained from cultured lymphocytes of the patient and a normal control with RNeasy Protect Mini Kit (Qiagen, Hilden, Germany), following the manufacturer’s instructions. *NAGS* cDNA was produced through RT-PCR using the specific *NAGS* reverse primer 7r (5’-ctggggctgctttctcct-3’) and the TaqMan Reverse Transcription kit (Applied Biosystems). Since the *NAGS* gene is poorly expressed in lymphocytes, the *NAGS* cDNA was firstly amplified with the primer pair Afw (5’-taagagccccccagtgcc-3’) and 7r in a fragment of 1815 bp, covering the entire coding region (exons 1–7) of the *NAGS* gene. Then, a fragment of 536 bp containing the c.344C>T (p.Ala115Val) variant and including exon 1 and part of exon 2, was amplified by nested PCR with A_1_fw (5’-cgctccagacagactgcc-3’) and Brev (5’-aaggccagggcaaaggcc-3’) primers. PCR product was checked by agarose gel electrophoresis and the relevant fragment was sequenced.

## 5. Conclusions

This report aims to raise clinical awareness in considering headaches as a presenting symptom of UCDs. Headaches in UCD patients are often recurrent, associated with behavioural symptoms, nausea and vomiting, or a self-selected low protein diet, and are due to hyperammonemia and/or hypoargininemia. Rapid ammonia measurement is crucial to allow early diagnosis and treatment of UCDs and their related headaches. Molecular analysis is the method of choice in making differential UCD diagnosis.

## Supplementary Materials

Supplementary materials can be found at http://www.mdpi.com/1422-0067/19/2/345/s1.

## Abbreviations

NAGSD	*N*-acetylglutamate synthase deficiency;
UCD	Urea cycle disorder;
NAGS	*N*-acetylglutamate synthase;
CPS1	Carbamoylphosphate synthetase 1;
hNAGS	Human NAGS;
AAK	Amino acid kinase;
NAT	*N*-acetyltransferase;
CPS1D	CPS1 deficiency;
NCG	*N*-carbamylglutamate;
ICHD-3 beta	International Classification of Headache Disorders 3rd edition, beta version;
EEG	Electroencephalogram;
n.v.	Normal values
CADASIL	Cerebral autosomal dominant arteriopathy with subcortical infarcts and leukoencephalopathy;
OTCD	OTC deficiency;
NO	Nitric oxid;
NSAIDs	Non-steroidal anti-inflammatory drugs;
HGMD	Human Gene Mutation Database;
gnomAD	Genome aggregation database;
RONN	Regional Order Neural Network;
RT-PCR	Reverse transcription polymerase chain reaction.
