# Supplementary material for: Late-Onset N-Acetylglutamate Synthase Deficiency: Report of a Paradigmatic Adult Case Presenting with Headaches and Review of the Literature
Source: Int J Mol Sci. 2018 Jan 24;19(2):345. doi: 10.3390/ijms19020345 (PMC5855567; doi:10.3390/ijms19020345)
Supplement: Supplementary file 1 [file ijms-19-00345-s001.pdf]

Table S1. Characteristics of headache in the patient based on a retrospective questionnaire.

**Starting age**	11 years, coinciding with menarche
**Family history for headaches**	Negative
**Initial location of pain**	Frontal region between the eyes
**Successive location of pain**	Frontal region
**Type of pain**	Pulsating and continuous
**Average pain level (1–10 scale)**	7
**Worst pain level (1–10 scale)**	10
**Average frequency**	Three to four headaches per week
**Frequency of the most severe bouts**	Four to five headaches per month
**Average duration**	Not determinable because the patient took analgesics within 1–2 hours from the onset of pain
**Time of occurrence during the day**	Variable
**Seasonality**	Not significant, even if headaches were more frequent after sun exposure
**Triggering factors**	Stress, meal consumption, menstrual cycle, pregnancy, insomnia, sun exposure and bright lights
**Impact on quality of life**	Important impact on daily activities without prompt analgesic treatment
**Pre-headache symptoms**	Cenesthesic symptoms, amaurosis fugax (one time) and sleep disorder
**Associated symptoms during the bout**	Increased sensitivity to light, nausea, vomiting, tachycardia, poor concentration, confusion and behavioral changes
**Improvement factors**	Darkness and analgesics
**Drugs for acute pain**	Acetylsalicylic acid; Ibuprofen

Table S2. Main results of biochemical tests performed in the patient during the last acute episode (52 years of age) after initiation of arginine therapy.

**Plasma Metabolite**	**Measured Value** (μmol/L)	**Normal Value** (mol/L)
Citrulline	30	17–53
Glutamine	809	399–823
Arginine	197	38–135
Ornithine	266	25–159
Alanine	584	206–498
**Urine metabolite**	**Measured Value** (mmol/mol creatinine)	**Normal Value** (mmol/mol creatinine)
Citrulline	8	0–4
Glutamine	65	20–76
Arginine	764	0–5
Ornithine	314	0–5
Alanine	56	16–68
Orotic acid	0.74	0.2–1.1

Plasma citrulline and glutamine were normal; plasma arginine, ornithine and alanine were conversely increased. Urinary glutamine, alanine and orotic acid were normal; increased urinary excretion of citrulline, arginine and ornithine was also detected. These findings were inconclusive for UCD diagnosis but could be compatible with a UCD during treatment.
